# Loss of Function of Rice Plastidic Glycolate/Glycerate Translocator 1 Impairs Photorespiration and Plant Growth

**DOI:** 10.3389/fpls.2019.01726

**Published:** 2020-01-24

**Authors:** Su-Hyeon Shim, Sang-Kyu Lee, Dae-Woo Lee, Dominik Brilhaus, Guangxi Wu, Sooyeon Ko, Choon-Hwan Lee, Andreas P.M. Weber, Jong-Seong Jeon

**Affiliations:** ^1^ Graduate School of Biotechnology and Crop Biotech Institute, Kyung Hee University, Yongin, South Korea; ^2^ Institute of Plant Biochemistry, Cluster of Excellence on Plant Sciences, Heinrich Heine University, Düsseldorf, Germany; ^3^ Department of Molecular Biology, Pusan National University, Busan, South Korea

**Keywords:** OsPLGG1, photorespiration, photosynthesis, plastidic glycolate/glycerate translocator, rice

## Abstract

Ribulose-1,5-bisphosphate carboxylase/oxygenase, the key enzyme of photosynthetic carbon fixation, is able to accept both O_2_ and CO_2_ as substrates. When it fixes O_2_, it produces 2-phosphoglycolate, which is detoxified by photorespiration and recycled to the Calvin–Benson–Bassham cycle. To complete photorespiration, metabolite transport across three organelles, chloroplasts, peroxisomes, and mitochondria, is necessary through transmembrane transporters. In rice (*Oryza sativa*) little is known about photorespiratory transmembrane transporters. Here, we identified the rice plastidic glycolate/glycerate translocator 1 (OsPLGG1), a homolog of *Arabidopsis* PLGG1. OsPLGG1 mutant lines, *osplgg1-1*, *osplgg1-2*, and *osplgg1-3*, showed a growth retardation phenotype, such as pale green leaf, reduced tiller number, and reduced seed grain weight as well as reduced photosynthetic carbon reduction rate due to low activities of photosystem I and II. The plant growth retardation in *osplgg1* mutants was rescued under high CO_2_ condition. Subcellular localization of OsPLGG1–GFP fusion protein, along with its predicted N-terminal transmembrane domain, confirmed that OsPLGG1 is a chloroplast transmembrane protein. Metabolite analysis indicated significant accumulation of photorespiratory metabolites, especially glycolate and glycerate, which have been shown to be transported by the *Arabidopsis* PLGG1, and changes for a number of metabolites which are not intermediates of photorespiration in the mutants. These results suggest that OsPLGG1 is the functional plastidic glycolate/glycerate transporter, which is necessary for photorespiration and growth in rice.

## Introduction

Ribulose-1,5-bisphosphate carboxylase/oxygenase (Rubisco) fixes the carbon from CO_2_ to carboxylate ribulose 1,5-bisphosphate (RuBP) during photosynthesis. After the oxygen contents of the atmosphere increased as a consequence of oxygenic photosynthesis by cyanobacteria, algae, and plants, O_2_ began to compete with CO_2_ for binding to Rubisco, which has binding affinity to both CO_2_ and O_2_. During fixation of O_2_ the toxic compound 2-phosphoglycolate (2-PG) is produced (Anderson, 1971), which is detoxified to 3-phosphoglycerate (3-PGA) by photorespiration and recycled to the Calvin–Benson–Bassham cycle. Thus, although photorespiration is considered as a wasteful pathway, it comprises the second largest carbon flux followed by photosynthesis in nature (Sharkey, 1988).

The photorespiration pathway is partitioned across multiple organelles, namely chloroplasts, peroxisomes, mitochondria, and the cytosol. In chloroplasts, especially in the stroma, 2-PG phosphatase (PGP) hydrolyzes 2-PG to glycolate, which is exported to peroxisomes across the membranes. Glycolate oxidase (GOX) irreversibly oxidizes glycolate to glyoxylate in peroxisomes, whereas H_2_O_2_ as a byproduct is decomposed by catalase (CAT). Then, two enzymes, serine:glyoxylate aminotransferase (SGT) and glutamate:glyoxylate aminotransferase (GGT) independently transaminate glyoxylate to glycine. As the amino donors, SGT more likely uses serine and GGT uses glutamate or alanine with a preference of glutamate over alanine. Glycine then moves to mitochondria where it is converted to serine, CO_2_, and ammonia through a combined reaction of deamination and decarboxylation by serine hydroxymethyltransferase (SHMT) and glycine decarboxylase (GDC). Serine is transported back to peroxisomes where the amino group is donated to glyoxylate by SGT and hydroxypyruvate is produced. The hydroxypyruvate is then reduced to glycerate by hydroxypyruvate reductase (HPR). Glycerate goes back to chloroplasts and is phosphorylated by glycerate kinase (GLYK) to produce 3-PGA, which is finally recycled to RuBP. In this pathway, one molecule of CO_2_ is released and three carbons are recycled to the Calvin–Benson–Bassham cycle from two molecules of 2-PG (Ogren, 1984; Bauwe et al., 2010; Eisenhut et al., 2019).

Since photorespiration occurs in different organelles, numerous membrane transport steps are necessary for function of the pathway (Reumann and Weber, 2006; Peterhansel et al., 2010). Although the photorespiratory pathway itself and its required enzymes have been well characterized, relatively little is known about the transport proteins that catalyze the metabolic flux between the involved compartments. For instance, in *Arabidopsis* the plastidic DiT1 and DiT2 were firstly identified as 2-oxoglutarate/malate translocator and glutamate/malate translocator, respectively, which are involved in nitrogen recycling during photorespiration (Somerville and Ogren, 1983; Somerville and Somerville, 1985; Renné et al., 2003; Schneidereit et al., 2006). A mitochondrial transporter A BOUT DE SOUFFLE (BOU) transports glutamate necessary for proper glycine decarboxylase activity (Eisenhut et al., 2013; Porcelli et al., 2018). It was recently proposed that *Arabidopsis* UCP1 and UCP2, homologs of the mammalian uncoupling protein 1, catalyze aspartate/glutamate exchange across the mitochondrial membrane and contribute to the export of reducing equivalents from the mitochondria in photorespiration (Monné et al., 2018). Earlier publications assumed that the *Arabidopsis* plastidic glycolate/glycerate transporter 1 (PLGG1) is involved in chloroplast development or functions against cell death due to the visible mutant phenotype of chlorotic leaves in which chloroplasts are destroyed (Yamaguchi et al., 2012; Yang et al., 2012). Later, PLGG1 was demonstrated to move two molecules of glycolate and one molecule of glycerate from/to chloroplasts, respectively (Pick et al., 2013). Thus, the visible symptom in *plgg1* mutants is due to the accumulation of toxic concentrations of glycolate and glycerate. In addition, the *Arabidopsis* bile acid sodium symporter BASS6 was found to export glycolate from chloroplasts. Double mutants of PLGG1 and BASS6 showed an additive growth defect, an increase in glycolate accumulation, and reductions in photosynthetic rates compared with either single mutant (South et al., 2017).

To improve plant growth and biomass production by reducing photorespiration, synthetic pathways which bypass the original photorespiratory pathway have been created in transgenic plants (Kebeish et al., 2007; Maier et al., 2012; Shen et al., 2019; South et al., 2019). For instance, in *Arabidopsis* introduction of five genes which encode glycolate dehydrogenase, glyoxylate carboligase, and tartonic semialdehyde reductase from *Escherichia coli* resulted in increased biomass production (Kebeish et al., 2007). In rice, transgenic plants expressing OsGLO3, OsOXO3, and OsCATC encoding glycolate oxidase, oxalate oxidase, and catalase, respectively, that are directed into rice chloroplasts by the rice Rubisco small subunit (rbcS) transit peptide, displayed significant increase in their growth and biomass (Shen et al., 2019). Also, it was shown that applying an artificial pathway which involves targeting of pumpkin malate synthase and chlamydomonas glycolate dehydrogenase to chloroplasts in tobacco plants in which PLGG1 was down-regulated by antisense RNA increased biomass by 40% compared with wild type (WT) (South et al., 2019).

In this study, we identified rice OsPLGG1, an *Arabidopsis* AtPLGG1 homolog, determined its subcellular localization, and characterized OsPLGG1 mutants morphologically, physiologically, and biochemically. Our results demonstrate that OsPLGG1 functions as a plastidic glycolate/glycerate transporter. It is the first characterized membrane transporter functioning in photorespiration in rice.

## Materials and Methods

### Plant Materials

*Japonica* WT [cultivar (cv.) Dongjin] and all the mutant seeds were germinated and grown on 1/2 Murashige and Skoog (MS) media for 10 days, and subsequently transferred to soil. Rice plants were grown either in a greenhouse with a light/dark cycle of 14/10 h (30/20°C) or in an experimental field plot under natural environmental conditions in summer (Eom et al., 2011). To observe the phenotype of mutants under high CO_2_ condition, WT and *osplgg1* mutants were grown with a light/dark cycle of 14/10 h (30/25°C) under either ambient or 0.3% CO_2_ condition in an environmental chamber.

### Bioinformatics Analysis

The *Arabidopsis* Information Resource (TAIR, https://www.arabidopsis.org/) was used to obtain the genomic and amino acid sequences of AtPLGG1 (At1g32080). To determine the candidate genes in rice, the BLAST tool of NCBI (https://blast.ncbi.nlm.nih.gov/Blast.cgi) was used. To predict cDNAs, all available EST and cDNA data from NCBI as well as MSU Rice Genome Annotation Project version 7 were used.

### Molecular Analysis of T-DNA Insertion Mutant Lines

T-DNA mutants for OsPLGG1 and OsPLGG2 were identified from our T-DNA insertional mutant pool (Rice Functional Genomic Express Database; http://signal.salk.edu/cgi-bin/RiceGE). Lines 1A22337, 2D21473, and 3A12105 were chosen for *osplgg1-1*, *osplgg2-1*, and *osplgg2-2* mutants (cv. Dongjin), respectively (Jeon et al., 2000; Jeong et al., 2002; Jeong et al., 2006). To isolate the homozygous T-DNA mutant lines for OsPLGG1 and OsPLGG2, genomic DNA PCR analysis was performed with primers G1F, G1R, G2F, G2R, T1, T2, and T3 (**Table S1**). Primer pairs G1F/G1R and G2F/G2R were used to detect the WT copy. Primer pairs T1/G1R, T2/G2R, and T3/G2R were used for T-DNA insertion mutant alleles of *osplgg1-1*, *osplgg2-1*, and *osplgg2-2*, respectively. *OsPLGG1* and *OsPLGG2* transcript abundances were measured in the respective mutants *via* quantitative RT-PCR using primer pairs RT1F/RT1R and RT2F/RT2R, respectively (**Table S1**). Rice *Ubiquitin5* (Os*UBQ5*; *LOC_Os01g22490*) was used as a loading control with primers UBQ5F/UBQ5R (**Table S1**).

### Expression of GFP Fusion Protein in Maize Protoplast

To determine subcellular localization of OsPLGG1, the *OsPLGG1* full-length cDNA without stop codon was amplified with primer pairs FLCF/FLCR (**Table S1**). The resulting cDNA was fused in-frame to the *Green Fluorescence Protein* (*GFP*) gene by inserting it between the *CaMV35S* promoter and *GFP* of a binary vector pH7FWG2 (Karimi et al., 2005). The constructed vector was transformed into maize protoplasts (Cho et al., 2009). GFP was visualized by excitation with the 488 nm line of the argon laser and a capturing emission at 522 nm by laser-scanning confocal microscopy (LSM 510 META; Carl Zeiss, Jena, Germany).

### Creation of CRISPR/Cas9 Mutant Lines

To determine an effective protospacer adjacent motif (PAM) and avoid any off-target for CRISPR-Cas9, the CRIPRdirect program (https://crispr.dbcls.jp/; Naito et al., 2015) and Centroidfold (http://rtools.cbrc.jp/centroidfold/) program were used. Two guide RNAs (5′-CCTCTTCTACGTCCCTTCCC-3′ and 5′-ACTGTACTGGGATACATGGT-3′) for *osplgg1-2* and *osplgg1-3*, respectively, were cloned to the pOs-sgRNA vector (Miao et al., 2013). Each of the resulting vectors was cloned into a destination vector, pH-Ubi-cas9-7, using the Gateway™ system (Miao et al., 2013) and then introduced into WT (cv. Dongjin) using *Agrobacterium-*mediated transformation (Jeon et al., 2000). The mutations of target sites were detected by sequencing of the amplified PCR products with primer pairs MF/MR (**Table S1**).

### Analysis of Photosynthetic Rate

Net photosynthetic carbon reduction activity was measured on the uppermost fully expanded leaves of 1-month-old rice plants grown in the paddy field using a portable gas-exchange system (Li-6400; Li-Cor, Lincoln, NE) at 1200 µmol m^−2^ s^−1^ photon flux density, leaf temperature of 25℃, and CO_2_ concentration of 350 µmol mol^−1^. Four biological replicates with three technical replicates each were tested for this analysis. Student's *t*-test was performed to show statistically significant differences.

### Analysis of Chlorophyll Fluorescence

*In vivo* chlorophyll fluorescence kinetics were measured using dark-adapted (overnight) leaves of 1-month-old plants grown in the paddy field using an IMAGING-PAM M-Series chlorophyll fluorometer (Walz, Effeltrich, Germany). F_o_ delineating the minimum fluorescence was determined after turning on the measuring light, and F_m_ delineating the maximum fluorescence was determined during illumination with an 0.8 s saturating pulse using dark-adapted leaves. F_m_′ delineating the maximum fluorescence was measured using leaves under actinic light illumination, and F delineating the fluorescence level was measured just before the determination of F_m_′. The photochemical efficiency of photosystem (PS) II or Fv/Fm = (F_m_ − F_o_)/F_m_; the effective PS II quantum yield Y(II) = (F_m_′ − F)/F_m_′; the quantum yield of non-regulated energy dissipation, Y(NO) = F/F_m_; and the quantum yield of regulated energy dissipation, Y(NPQ) = (F/F_m_′) − (F/F_m_); these equations were used during analyzing (Klughammer and Schreiber, 2008a). For high light stress, leaf segments were illuminated either in 900 µmol m^−2^ s^−1^ for 20 min or 1,800 µmol m^−2^ s^−1^ for 3 h. Fv/Fm was measured after dark-adaptation for 15 min.

### Analysis of P700 Absorption

P700 was determined as described by Klughammer and Schreiber (2008b) with slight modifications using a pulse amplitude modulated fluorometer (PAM101/102/103, Walz, Effeltrich, Germany). Quantum yield of photochemical energy conversion of PS I, Y(I) = (Pm′ − P)/(Pm − Po); quantum yield of non-photochemical energy dissipation due to donor-side limitation of PS I, Y(ND) = (P − Po)/(Pm − Po); and quantum yield of non-photochemical energy dissipation due to acceptor-side limitation of PS I, Y(NA) = (Pm − Pm′)/(Pm − Po); these equations were used during analyzing. In these equations, Po is the zero P700 signal level, P is the intermediate P700 signal after illumination of actinic light (120 μmol photons m^−2^ s^−1^) for 5 min, Pm′ is the maximal P700 signal measured during actinic light illumination with an 0.8 s saturating pulse, and Pm is the maximal P700 signal measured again after full oxidation of P700 by illuminating far-red light.

### Metabolite Analysis Using Gas Chromatography Mass Spectrometry (GC–MS)

Uppermost fully expanded leaves of 1-month-old WT and *osplgg1* mutant plants grown in the paddy field were collected in the middle of the day, immediately frozen in liquid nitrogen, and stored at −80°C until further processing. Metabolites were analyzed *via* gas chromatography–mass spectrometry (GC–MS) using protocols adapted from Lisec et al. (2006) and Gu et al. (2012). Metabolites were extracted from 40–70 mg fresh weight of ground leaf materials using 1.5 ml CHCl_3_/CH_3_OH/H_2_O (1:2.5:1, v/v) mixture including 5 µM ribitol as internal standard pre-cooled to −20°C, then mixed on a rotator for 10 min and centrifuged at 20,000 g for 2 min at 4°C. A total of 50 µl of the supernatant were dried completely in a vacuum concentrator and derivatized in two steps *via* an MPS-Dual-head autosampler (Gerstel): (1) with 10 µl methoxyamine hydrochloride [Acros organics; freshly prepared at 20 mg ml^−1^ in pure pyridine (Sigma-Aldrich)] and shaking at 37°C for 90 min, (2) adding 90 µl N-Methyl-N-(trimethylsilyl)trifluoroacetamide (MSTFA; Macherey-Nagel) and shaking at 37°C for 30 min. After incubation for 2 h at room temperature, 1 µl of derivatized compounds was injected at a flow of 1 ml min^−1^ with an automatic liner exchange system in conjunction with a cold injection system (Gerstel) in splitless mode (ramping from 50 to 250°C at 12°C s^−1^) into the GC. Chromatography was performed using a 7890B GC system (Agilent Technologies) with a HP-5MS column with 5% phenyl methyl siloxane film (Agilent 19091S-433, 30 m length, 0.25 mm internal diameter, 0.25 µM film). The oven temperature was held constant at 70°C for 2 min and then ramped at 12.5°C min^−1^ to 320°C at which it was held constant for 5 min; resulting in a total run time of 27 min. Metabolites were ionized with an electron impact source at 70 V and 200°C source temperature and recorded in a mass range of m/z 60 to m/z 800 at 20 scans per sec with a 7200 GC-QTOF (Agilent Technologies). Metabolites were identified *via* MassHunter Qualitative (v b06, Agilent Technologies) by comparison of spectra to the NIST14 Mass Spectral Library (https://www.nist.gov/srd/nist-standard-reference-database-1a-v14). A standard mixture containing all target compounds at a concentration of 50 µM was processed in parallel to the samples as a response check and retention time reference. Peaks were integrated using MassHunter Quantitative (v b06, Agilent Technologies). For relative quantification, all metabolite peak areas were normalized to the corresponding fresh weight used for extraction and the peak area of the internal standard ribitol (Sigma-Aldrich). Three biological replicates with three technical replicates each were tested for this analysis. Student's *t*-test was performed to show statistically significant differences. Additional experimental details are provided in **Data Sheet S1** following reporting standards suggested by Fernie et al. (2011).

## Results

### Identification of AtPLGG1 Homologs in Rice

To identify the *PLGG1* candidate genes in the rice genome, we performed a BLAST search using the previously reported amino acid sequences for the AtPLGG1 (At1g32080) in *Arabidopsis*. We selected two candidates, named *OsPLGG1* (*LOC_Os01g32830*) and *OsPLGG2* (*LOC_Os10g42780*) from the NCBI database. While the annotation of *OsPLGG1* was incorrect [*MSU* Rice Genome *Annotation* (*Osa1*) *Release 7]*, the cDNA sequence (EEE54674.1) from a Chinese group appeared to correctly match that from our WT (cv. Dongjin) and aligned well with its homologs from many plant species (Yu et al., 2005; Wang and Bayles, 2013; Kang et al., 2016). Those two rice proteins showed identities of 76% and 69% to AtPLGG1, respectively (**Figure S1**). Heat map expression analysis of publicly available Affymetrix rice microarray data revealed leaf and shoot-preferential expression (**Figure S2**), suggesting their role in these organs/tissues.

Similar to AtPLGG1 which contains an N-terminal chloroplast targeting peptide followed by 12 transmembrane regions, SMART search (http://smart.embl-heidelberg.de/) indicated that OsPLGG1 and OsPLGG2 harbor 10 and 11 transmembrane helices, respectively. Kyoto Encyclopedia of Genes and Genomes search (KEGG, http://www.genome.jp/kegg/) also showed that like AtPLGG1, both contain LrgA and LrgB domains, components for bacterial cid/lrgAB operons which regulate bacterial programmed cell death and cell lysis (Brunskill and Bayles, 1996; Rice and Bayles, 2008). This suggests that OsPLGG1 and OsPLGG2 are likely membrane proteins. Moreover, TargetP Program (Emanuelsson et al., 2000; http://www.cbs.dtu.dk/services/TargetP) identified a predicted N-terminal chloroplast targeting peptide with 77 amino acids in OsPLGG1 but not in OsPLGG2. Thus, only OsPLGG1 appeared to retain the chloroplast targeting peptide that is absent in OsPLGG2 (**Figure S1**), suggesting that OsPLGG1 is a rice homolog of AtPLGG1, whereas OsPLGG2 may have a distinct function.

### Characterization of OsPLGG1 and OsPLGG2 Mutants

**Figure 1:**
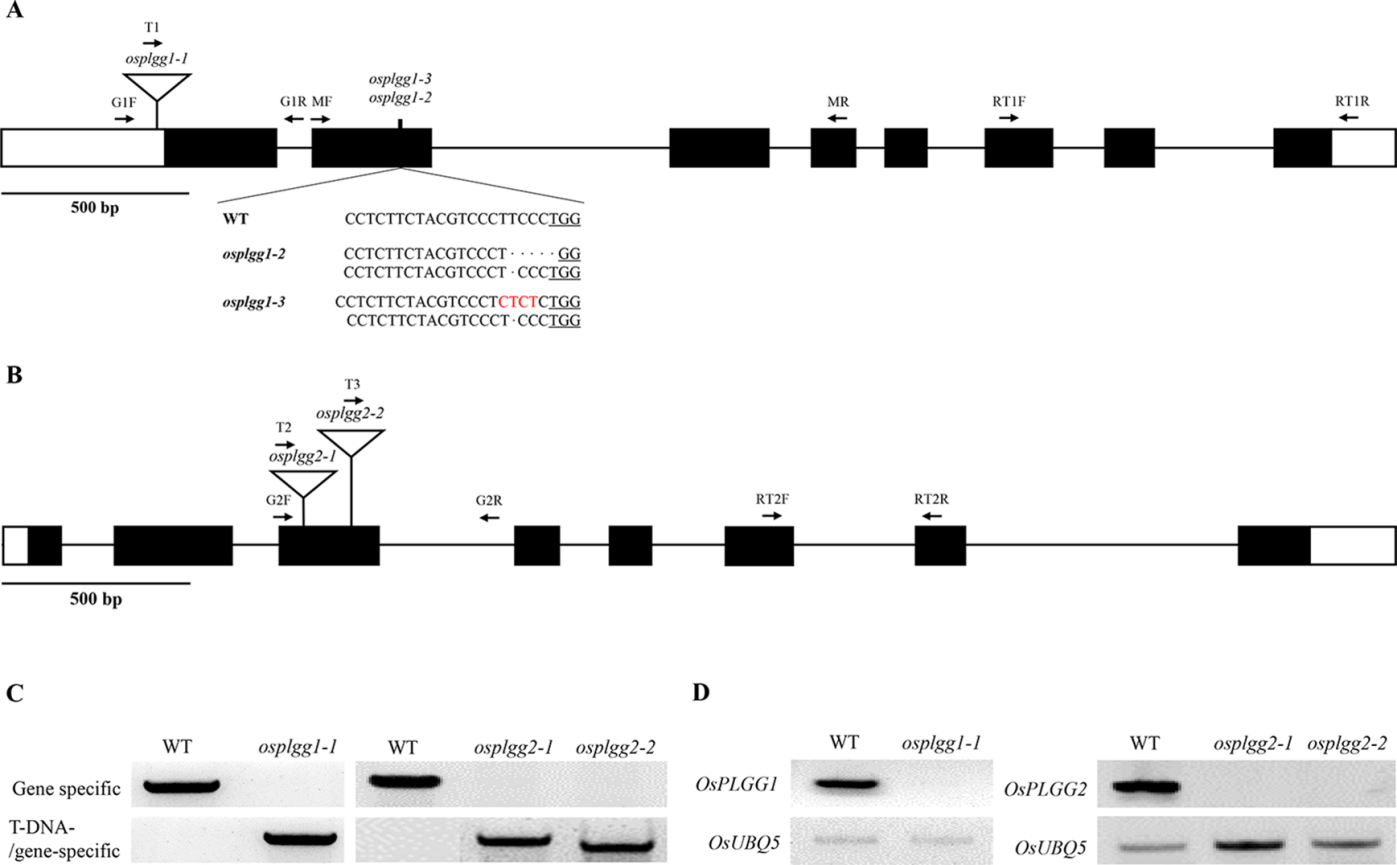
Characterization of the OsPLGG mutants. **(A)** Schematic representation of OsPLGG1 genomic structure with position of the T-DNA insertion (*osplgg1-1*) and Indel mutant alleles (*osplgg1-2* and *osplgg1-3*) created by CRISPR/Cas9 in cv. Dongjin. The underlined sequence is the PAM target site. **(B)** Schematic representation of OsPLGG2 genomic structure with position of the T-DNA insertions (*osplgg2-1* and *osplgg2-2*) in cv. Dongjin. Both *OsPLGG1* and *OsPLGG2* consist of eight exons (black boxes) and seven introns (line between the black boxes). Arrows indicate primer location for genomic DNA PCR and RT-PCR. **(C)** Genomic DNA PCR analysis of *osplgg1-1* (left) and *osplgg2-1* and *osplgg2-2* (right). Gene specific primers (upper) amplify WT and T-DNA/gene-specific primers (lower) T-DNA insertion alleles. **(D)** RT-PCR analysis of *osplgg1-1* (left) and *osplgg2-1* and *osplgg2-2* (right). *OsUBQ5* was used as an internal control.

To determine whether either OsPLGG1 or OsPLGG2 or both play a critical role in photorespiration, we isolated T-DNA insertion mutants for OsPLGG1 and OsPLGG2 from our T-DNA mutant population (Jeon et al., 2000; Jeong et al., 2002; Jeong et al., 2006). As a result, OsPLGG1 mutant, *plgg1-1* carried a T-DNA insertion in the 5′-UTR (**Figure 1A**) and OsPLGG2 mutants, *osplgg2-1* and *osplgg2-2* carried T-DNA insertions at different positions in the third exon (**Figure 1B**). All homozygous mutants were isolated from their segregating progeny by genotyping using genomic DNA PCR with gene- and T-DNA-specific primers (**Figure 1C**). RT-PCR confirmed null expression of *OsPLGG1* or *OsPLGG2* in the homozygous mutants (**Figure 1D**).

**Figure 2:**
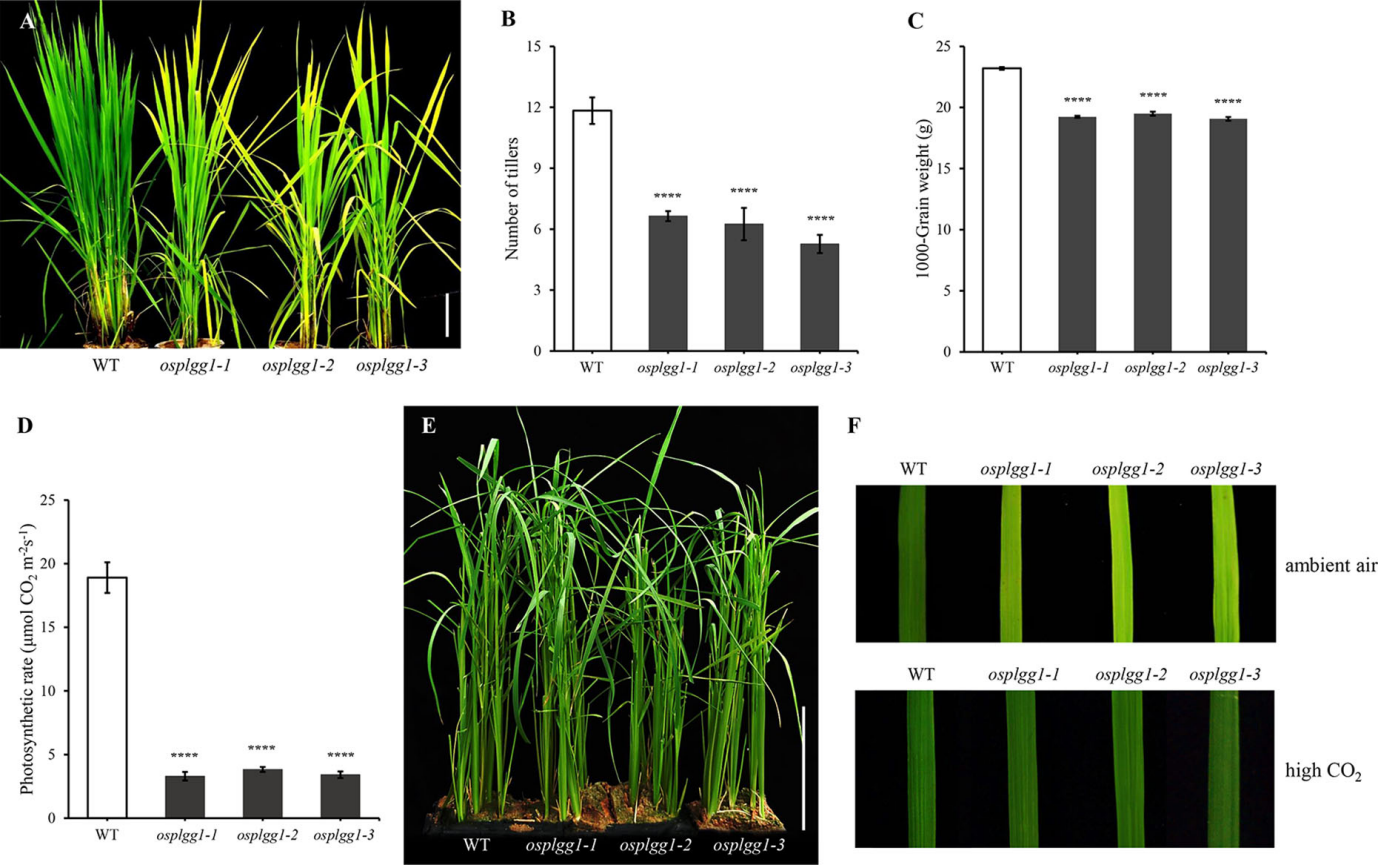
Phenotype analysis of *osplgg1* mutants. **(A)** Mature plants of WT, *osplgg1-1*, *osplgg1-2* and *osplgg1-3* mutants grown for 2 months in the paddy field. Bar = 10 cm. **(B, C)** Number of tillers **(B)** and 1000-grain weight **(C)** of WT, *osplgg1-1*, *osplgg1-2*, and *osplgg1-3* plants. The experiment consisted of five independent plants per each line. Error bars indicate the standard error of the mean (SEM). ****P < 0.0001. Student's *t*-test was used to show statistical differences. **(D)** Photosynthetic carbon reduction rate of WT, *osplgg1-1*, *osplgg1-2*, and *osplgg1-3* in leaves of 1-month-old WT and *osplgg1* mutant plants grown in the paddy field. Four independent biological replicates were used in the experiment, each having three technical replicates. Error bars indicate SEM. ****P < 0.0001. **(E)** Phenotypic recovery of *osplgg1-1*, *osplgg1-2*, and *osplgg1-3* mutant plants grown under high CO₂ condition for 1 month in an environmental growth chamber. Bar = 10 cm. **(F)** Leaf blades of WT, *osplgg1-1*, *osplgg1-2*, and *osplgg1-3* plants grown under ambient air (top) or high CO₂ condition (bottom) for 3 weeks in an environmental growth chamber.

Only *osplgg1-1* plants displayed pale green leaves from the early developmental stage and later the growth was severely retarded, yielding only a few tillers during maturation (**Figures 2A, B**). In *osplgg1-1*, the average number of tillers was about half (6.6) of WT (11.8) (**Figure 2B**), and 1000-grain weight was also reduced to 19.32 g compared to 23.37 g of WT (**Figure 2C**). Consistently with the pale green leaf, the photosynthetic carbon reduction rate was significantly reduced to 20% of WT in *osplgg1-1* (**Figure 2D**). This growth retardation of *osplgg1-1* mimicked that of *atplgg1* grown under ambient air condition (Pick et al., 2013). Retarded growth phenotype of *osplgg1-1* was rescued to levels comparable to WT under high CO_2_ (0.3%) in an environmental growth chamber (**Figures 2E, F** bottom), whereas the pale green leaves and growth retardation remained unchanged under ambient air condition (**Figure 2F** top). However, OsPLGG2 mutants, *osplgg2-1* and *osplgg2-2*, did not exhibit any visible growth phenotype (**Figure S3**).

To further confirm the growth retardation in *osplgg1-1*, we generated two additional OsPLGG1 mutant alleles *via* the CRISPR/Cas9 system. The PAM site in the second exon was selected as the target site. Among more than 30 homozygous mutant lines of OsPLGG1, only 10 survived as early growth of most of the primary mutant plants was very poor. We randomly selected two biallelic mutant plants for further experiments and designated them as *osplgg1-2* (T deletion and TCCCT deletion relative to WT) and *osplgg1-3* (T deletion and TCC to CTCT substitution) (**Figure 1A**). We predicted that T deletion and TCCCT deletion in *osplgg1-2* induce severe changes after the 180th amino acid and premature stop codons at the 221st and the 232nd amino acid, respectively. In *osplgg1-3*, T deletion and TCC to CTCT substitution also cause severe changes after the 180th amino acid and premature stop codons at the 221st and 233rd amino acid, respectively.

*Osplgg1-2* and *osplgg1-3* both showed the same morphological phenotype with *osplgg1-1.* They displayed pale green leaves and growth retardation and had fewer tillers (**Figures 2A, B**). Similar to *osplgg1-1*, *osplgg1-2* and *osplgg1-3* had only five to six tillers (**Figure 2B**) and reduced 1000-grain weight (**Figure 2C**). We also found a similarly great reduction of the photosynthetic carbon reduction rate in *osplgg1-2* and *osplgg1-3* (**Figure 2D**). *osplgg1-2* and *osplgg1-3* also rescued plant growth to levels comparable to WT under high CO_2_ (0.3%) in an environmental growth chamber, whereas the pale green leaves and growth retardation remained unchanged under ambient air condition (**Figures 2E, F**). This result supports that OsPLGG1 deficiency resulted in plant growth retardation in rice.

**Figure 3:**
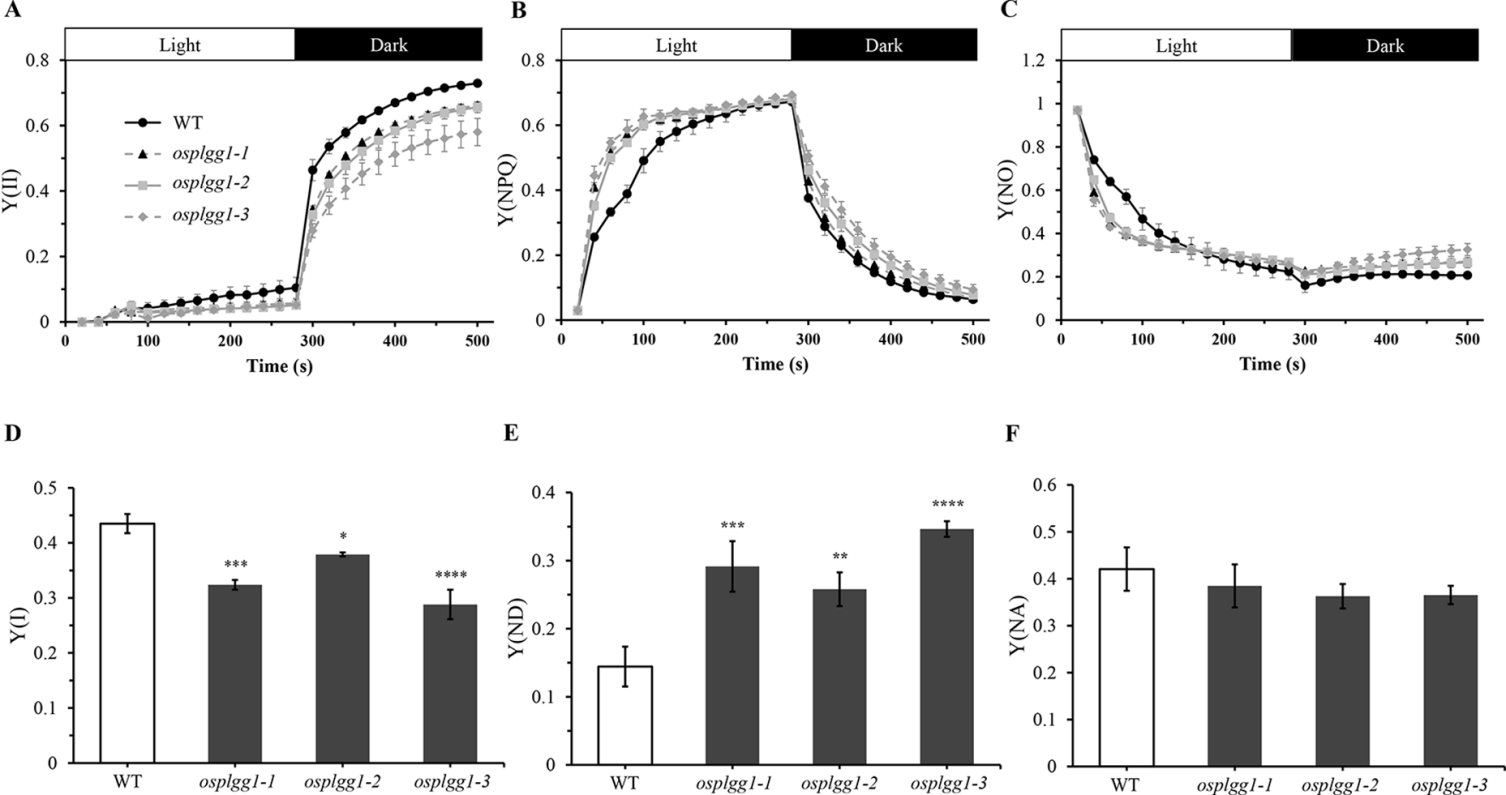
Analysis of PS II and PS I activities of *osplgg1* mutants, *osplgg1-1*, *osplgg1-2*, and *osplgg1-3*. **(A)** Effective photochemical quantum yield of PS II, Y(II). **(B)** Quantum yield of regulated energy dissipation of PS II, Y(NPQ). **(C)** Quantum yield of non-regulated energy dissipation of PS II, Y(NO). Detached leaves were illuminated with actinic light (550 µmol m^−2^ s^−1^) for 280 s, followed by recovery in darkness for 280 s. Saturation pulse was applied in every 20 s. **(D)** Quantum yield of photochemical energy conversion in PS I, Y(I). **(E)** Quantum yield of non-photochemical energy dissipation due to donor-side limitation of PS I, Y(ND). **(F)** Quantum yield of non-photochemical energy dissipation due to acceptor-side limitation of PS I, Y(NA). Detached leaves were illuminated with actinic light (120 µmol m^−2^ s^−1^) for 5 min for the activation of Calvin–Benson–Bassham cycle before the measurement of maximal P700 signals. Three independent biological replicates were used in the experiment, each having four technical replicates. Error bars indicate SEM. Student's *t*-test was used to show statistical differences. *P < 0.05; **P < 0.01; ***P < 0.001; **** < 0.0001.

The great reduction in photosynthetic carbon fixation rates per leaf area in OsPLGG1 mutants (**Figure 2D**) can partly be explained by the inactivation of PS I and/or PS II. Experiments under two different photoinhibitory illumination conditions (900 µmol m^−2^ s^−1^ for 20 min or 1,800 µmol m^−2^ s^−1^ for 3 h) revealed that PS IIs of OsPLGG1 mutants, *osplgg1-1*, *osplgg1-2*, and *osplgg1-3*, were very sensitive to high light stress, and PS IIs of the mutants were partly damaged even after overnight dark-adaptation (**Table 1**). Under actinic light illumination, the effective photochemical quantum yield of PS II, Y(II), was low and its recovery under darkness was slow in the mutants (**Figure 3A**). The *osplgg1* mutants showed fast Y(NPQ) development kinetics in the light and slow decay kinetics in darkness (**Figure 3B**), and these differences were compensated in Y(NO), because the sum of these three parameters is equal to 0 (**Figure 3C**). The effective photochemical quantum yield of PS I, Y(I), was also low in the mutants (**Figure 3D**), and the low activities of PS II and PS I resulted in higher donor-side limitation, Y(ND), in the mutants compared with WT (**Figure 3E**). However, there was no significant difference in the degree of acceptor-side limitation, Y(NA), between WT and mutants (**Figure 3F**).

**Table 1:** Effects of two different photoinhibitory illumination on Fv/Fm in WT and *osplgg1* mutants.

	Dark	900 µmol m^−2^ s^−1^	1,800 µmol m^−2^ s^−1^
WT	0.779 ± 0.001	0.724 ± 0.017	0.451 ± 0.067
*osplgg 1-1*	0.701 ± 0.013	0.583 ± 0.042^***^	0.300 ± 0.058^*^
*osplgg 1-2*	0.704 ± 0.032	0.610 ± 0.009^**^	0.274 ± 0.039^*^
*osplgg 1-3*	0.617 ± 0.046^***^	0.573 ± 0.023^***^	0.231 ± 0.038^**^

Plants were overnight dark-incubated, and leaf segments were illuminated either in 900 µmol m^−2^ s^−1^ for 20 min or 1,800 µmol m^−2^ s^−1^ for 3 h. Photosynthetic efficiency of PS II, Fv/Fm, was measured using leaf segments of WT and *osplgg1* mutants after dark-adaptation for 15 min. Four independent biological replicates were used in the experiment, each having four technical replicates. Values are means ± SD. Student's *t*-test was used to show statistical differences. *P < 0.05; **P < 0.01; ***P < 0.001.

### Subcellular Localization of OsPLGG1

**Figure 4:**
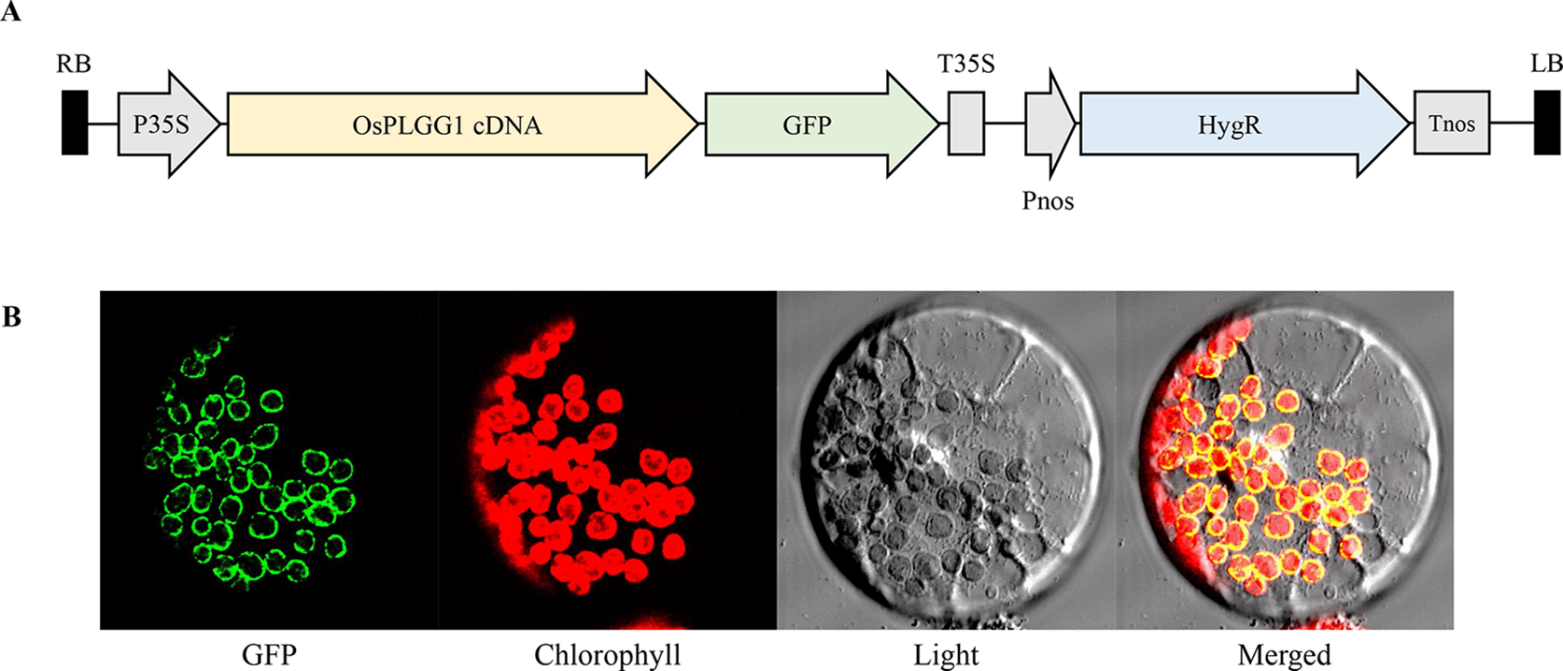
Subcellular localization of OsPLGG1-GFP in maize protoplast. **(A)** Schematic diagram of OsPLGG1-GFP fusion construct. P35S, *CaMV35S* promoter; T35S, *CaMV35S* terminator; Pnos, *Nopaline synthase* promoter; Tnos, *Nopaline synthase* terminator. HygR, *Hygromycin phosphotransferase*. **(B)** GFP localization in maize protoplast. Fluorescent GFP signals, chlorophyll autofluorescence, light microscope view, and a merged image are shown from left to right.

According to the subcellular localization prediction, only OsPLGG1 retained an N-terminal chloroplast targeting peptide and its mutant mimicked the *atplgg1* phenotype. To determine its *in vivo* subcellular localization, a full-length *OsPLGG1* cDNA without stop codon was fused in-frame to *GFP* gene under the control of the *CaMV35S* promoter of a plant expression vector (**Figure 4A**). The resulting construct was transiently expressed in maize protoplasts. The green fluorescence signal observed by microscopy clearly overlapped with the outer layer of chlorophyll autofluorescence. This result supports the chloroplast membrane localization of OsPLGG1 (**Figure 4B**).

### Changes in Photorespiratory Metabolites in *Osplgg1* Mutants

**Figure 5:**
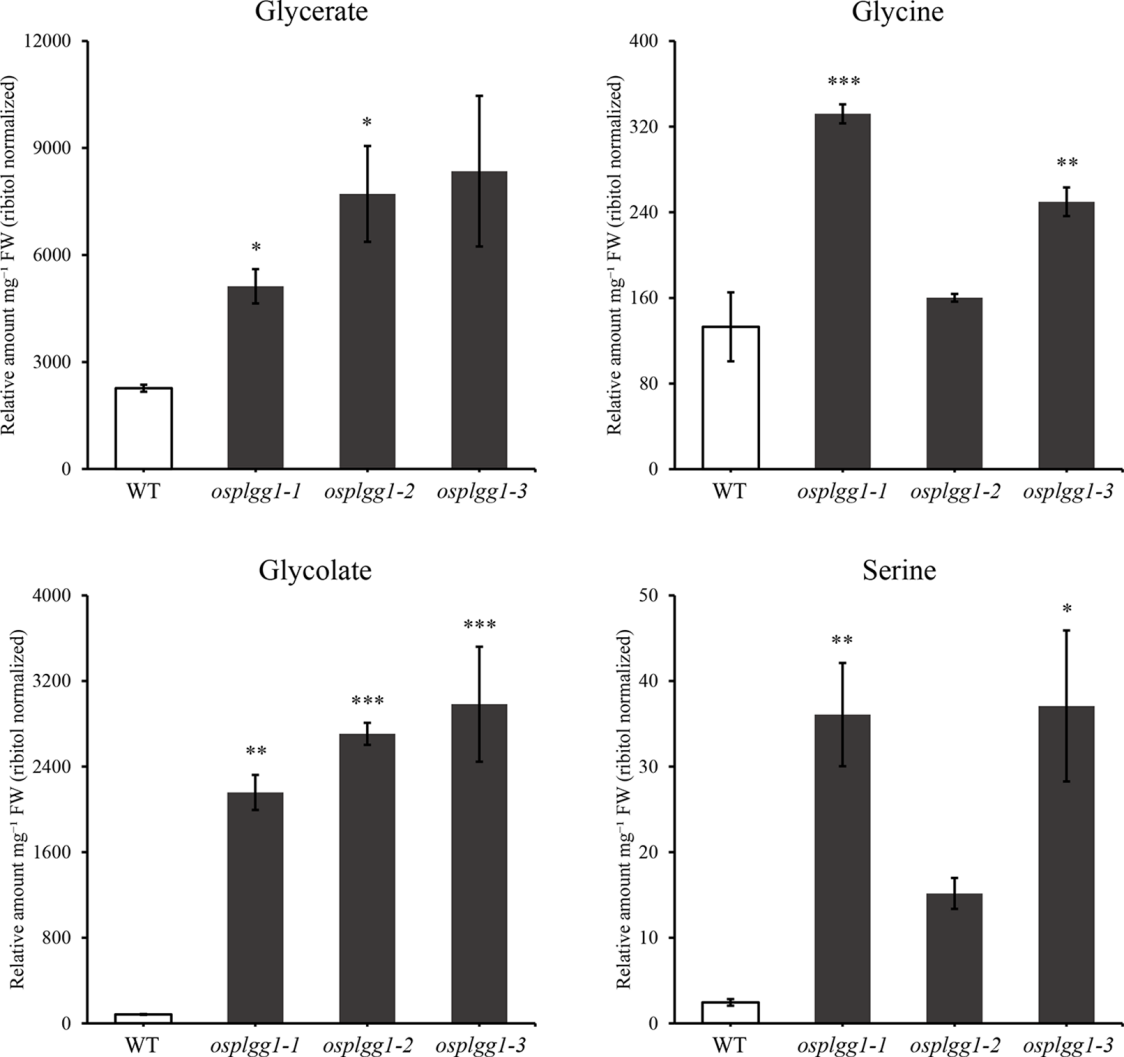
Contents of representative photorespiratory metabolites, glycerate, glycine, glycolate, and serine in leaves of 1-month-old WT and *osplgg1* mutant plants grown in the paddy field. The relative metabolite levels were normalized to an internal standard (ribitol) and the fresh weight of the samples. Three independent biological replicates were used in the experiment, each having three technical replicates. Error bars indicate SEM. Student's *t*-test was used to show statistical differences. *P < 0.05; **P < 0.01; ***P < 0.001.

To identify the role of OsPLGG1 in photorespiration, we analyzed photorespiratory metabolites in WT and three *osplgg1* mutants. Since photorespiration occurs only during the day, all leaf samples were harvested at the middle of the day. Most of the photorespiratory intermediates, including glycerate and glycolate, were highly accumulated in *osplgg1* mutants, which is well consistent with the observation in *atplgg1* (**Figure 5**). In the mutants, significantly increased abundances of the photorespiratory metabolites glycerate (2.6-, 3.41-, and 3.69-fold), glycolate (25.87-, 32.43-, and 35.74-fold), glycine (2.5-, 1.2-, and 1.88-fold), and serine (14.73-, 6.19-, and 15.14-fold) were determined relative to WT for *osplgg1-1*, *osplgg1-2*, and *osplgg1-3*, respectively (**Figure 5**).

### Differential Changes of Additional Metabolites Induced in *Osplgg1* Mutants

In *osplgg1* mutant leaves, we further analyzed a number of additional metabolites and found significant changes for some metabolites which are not intermediates of photorespiration (**Figure S4**). The levels of hydroxyglutarate, mannitol, and xylose were highly elevated, while those of GABA (*gamma*-aminobutyric acid), maltose, methionine, quinate, raffinose, shikimate, succinate, and tyrosine were significantly decreased in all three *osplgg1* mutants (**Figure S4**). Hydroxyglutarate, mannitol, and xylose were increased to average 8.6-, 2.5-, and 2.2-fold, respectively in *osplgg1* mutants. In contrast, *osplgg1* plants showed declines of 3.7-, 2.8-, 4.4-, 8.2-, 6.8-, 2.3-, 2.5-, and 12.8-fold each in GABA, maltose, methionine, quinate, raffinose, shikimate, succinate, and tyrosine compared with WT (**Figure S4**).

## Discussion

Photorespiration requires various transmembrane transporters residing in three organelles, chloroplasts, peroxisomes, and mitochondria, none of which have been identified in rice. Here, we report OsPLGG1 that functions as the plastidic glycolate/glycerate translocator in rice. *In silico* analysis of publicly available data proposed two candidates for the rice plastidic glycolate/glycerate translocator, OsPLGG1 and OsPLGG2 (**Figure S1**). Among the mutant plants for both genes, *osplgg1* mutants only displayed severe growth retardation with pale-green leaves, less tillers, lower grain weight as well as reduced photosynthesis ability (**Figures 2** and **3**), which mimics the bleached lesions on the leaf lamina and growth retardation of *Arabidopsis plgg1* mutant (Pick et al., 2013). The plant growth retardation in *osplgg1* mutants was rescued under high (0.3%) CO_2_ condition (**Figure 2**). These results proposed OsPLGG1 as a functional homolog of AtPLGG1. In contrast, OsPLGG2 mutants, *osplgg2-1* and *osplgg2-2*, did not show any visible phenotype (**Figure S3**). Chloroplast membrane localization was confirmed by detection of OsPLGG1-GFP in maize protoplasts (**Figure 4**). Metabolite analysis in *osplgg1* mutants revealed significant accumulation of photorespiratory metabolites including glycolate and glycerate that have been shown to be transported by the *Arabidopsis* PLGG1 (Pick et al., 2013) (**Figure 5**). Therefore, accumulated glycolate and glycerate resulted from the blocked OsPLGG1 transporter most likely support that OsPLGG1 functions as glycolate/glycerate translocator across the chloroplast membrane. We speculate that reduced consumption of 2-PG in *osplgg1* mutants leads to harmful effects on plants and eventually limited reduction of 2-PG to 3-PGA decreases the photosynthetic rate. In this regard, we could not observe any significant increase in the acceptor-side limitations in the mutants. Instead, there were significant decrease in effective photochemical quantum yield of PS II and PS I in the mutants (**Figure 3**). Changes in PS II photochemical efficiency or Fv/Fm revealed that PS II of the mutants was sensitive to photoinhibitory illumination (**Table 1**).

In *osplgg1* mutant leaves, we found increased relative abundances of the photorespiratory metabolites, glycine and serine, and also significant changes for a number of metabolites which are not intermediates of photorespiration (**Figure 5** and **Figure S4**). Photorespiration has been known to interact with associated processes such as nitrogen assimilation, one-carbon (C1) metabolism, sulfur metabolism, and the GABA shunt (Bauwe et al., 2010; Bloom et al., 2010; Peterhansel et al., 2010; Bauwe et al., 2012; Hodges et al., 2016; Obata et al., 2016; Samuilov et al., 2018; Eisenhut et al., 2019). Glycine decarboxylation by GDC releases ammonia and CO_2_ that are reassimilated. Thus, the accumulated glycine in *osplgg1* mutants affected nitrogen metabolism. Serine is supplied for the cytosolic production of C1-compounds that are in turn required for C1 metabolism, which is essential for the synthesis of nucleic acids, proteins, pantothenate, and methylated molecules (Hanson and Roje, 2001). It is also probable that the accumulated serine may affect sulfur metabolism including cysteine biosynthesis in *osplgg1* mutants. GABA, an intermediate of the GABA shunt pathway, was decreased in all three *osplgg1* mutants (**Figure S4**). In addition, our metabolite analysis suggests that some metabolites associated with the TCA cycle, the pentose phosphate pathway, starch pathway, methionine synthesis, and the shikimate pathway (**Figure S4**) were affected likely as compensation for retarded plant growth. Nevertheless, to better understand the core photorespiratory pathway and its metabolic interdependency, detailed investigation including kinetic analysis of important metabolites is a prerequisite in photorespiratory mutants.

Improvement of photosynthetic rate is one of the main approaches for increased crop productivity. In this regard, a synthetic route for photorespiratory bypass produces more biomass and leads to increased crop yield (Kebeish et al., 2007; Shen et al., 2019). Interestingly, when a synthetic glycolate catabolic pathway was introduced into chloroplasts of WT tobacco and a line displaying reduced expression of *PLGG1*, the latter showed significantly enhanced plant growth (South et al., 2019). Identification of photorespiratory membrane transporters is a prerequisite for similarly engineering photorespiratory bypass routes to improve yield potential in crop plants including rice that can contribute to feed an increasing world population. Therefore, the OsPLGG1 discovered here may provide a good opportunity to improve productivity in rice as well as to understand photorespiration in rice.

## Data Availability Statement

All datasets generated for this study are included in the article/**Supplementary Material**.

## Author Contributions

J-SJ conceived and designed the experiments. S-HS, S-KL, D-WL, GW, and SK performed the experiments. S-HS, S-KL, D-WL, DB, C-HL, AW, and J-SJ analyzed the data. S-HS, S-KL, DB, C-HL, AW, and J-SJ wrote the paper.

## Funding

This work was supported by a grant from the Next Generation BioGreen 21 Program of the Rural Development Administration of Korea (PJ013172). AW appreciates funding by the Deutsche Forschungsgemeinschaft (DFG, German Research Foundation) under Germany's Excellence Strategy—EXC-2048/1—Project ID: 390686111, and the Federal Ministry of Education and Research, Project FormatPlant (FKZ: 031B0194).

## Conflict of Interest

The authors declare that the research was conducted in the absence of any commercial or financial relationships that could be construed as a potential conflict of interest.
