# Supplementary material for: Loss of Function of Rice Plastidic Glycolate/Glycerate Translocator 1 Impairs Photorespiration and Plant Growth
Source: Front Plant Sci. 2020 Jan 24;10:1726. doi: 10.3389/fpls.2019.01726 (PMC6993116; doi:10.3389/fpls.2019.01726)
Supplement: Supplementary file 2 [file Image_1.pdf]

```
AtPLGG1     MATL--LATPIFSPLASSPARNRLSCSKIRFGSKNGKILNSDGAQKLNLSKFRKPDGQRF     58
OsPLGG1     MAATSSSSSPCMIASLRSSHRCRLSPSATATSPPRLRTLPPRRCRRNPSSSSSSSRQAAA     60
OsPLGG2     ------------------------------------------------------------     0

AtPLGG1     LQMGSS-KEMNFERKLSVQAMDGAGT-----GNTSTISRNVIAISHLLVSLGIILAADYF     112
OsPLGG1     ISMAPANPRHRLIAPIHAAAAAGGGGGGAGATSPSGLPSL-VGIAHLLVSLGIVLASDKF     119
OsPLGG2     --MAVDDLNHHLIA---------VGVGEEAATAHCGLRTLVRNMAQLVVSLGILVAADKL     49
              *.    . .:            *         . :      :::*:*****::*:* :

AtPLGG1     LKQAFVAASIKFPSALFGMFCIFSVLMIFDSVVPAAANGLMNFFEPAFLFIQRWLPLFYV     172
OsPLGG1     LKQAFAAASIKFPSALFGMFCVFSVLVVLDAFAPALAKGFMDFFEPATLFIQRWLPLFYV     179
OsPLGG2     VEQAFAAASIKFPSALFAMFCVFALLLF---LPPSLANGFMAFFDPATVFIHRWLPLFFV     106
            ::***.***********.***:*::*:.   . *: *:*:* **:** :**:******:*

AtPLGG1     PSLVVLPLSVRDIPAASGVKICYIVAGGWLASLCVAGYTAIAVRKMVKTEMTEAEPMAKP     232
OsPLGG1     PSLVVLPLAVRDVPAASGLKIFLIIFGGWFASLMVAGYTALTVRKIVQTQLIPAEPMSKP     239
OsPLGG2     PSLVVLPLAVRDVSPASALKILFITFGGWFASLVVAGYTALSVRRIVKTQLIPAEPMKRP     166
            ********:***:  **.:**  *  ***:*** ******::**::*:*::  **** :*

AtPLGG1     SPFSTLELWSWSGIFVVSFVGALFYPNSLGTSARTSLPFLLSSTVLGYIVGSGLPSSIKK     292
OsPLGG1     SPFATLEFWAWGAVFVASFAVAYVNPTALGTTARTCLPFLLASTVLGYMVGSGIPSGVKK     299
OsPLGG2     SPFGPLEFWAWAAVFVASFAVAYVSPTALGTTATTCLPFLLASTVFGYILGSRLPSGVKK     226
            ***. **:*:*..:**.**. * . *.:***:* *.*****:***:**::** :**.:**

AtPLGG1     VFHPIICCALSAVLAALAFGYASGSGLDPVLGNYLTKVASDPGAGDILMGFLGSVILSFA     352
OsPLGG1     VLHPIICCALSADLAAIAYGYLSRSGVDAVLGDYLTKAPSNPGAGDVLMGFLGSVIISFA     359
OsPLGG2     VLHPIICCALSADLAAVAYGYLSRSGVDAVLGDYLTESPSNPGAGDILMGFLGSVIISFA     286
            *:********** ***:*:** * **:* ***:***:  *:*****:*********:***

AtPLGG1     FSMFKQRKLVKRHAAEIFTSVIVSTVFSLYSTALVGRLVGLEPSLTVSILPRCITVALAL     412
OsPLGG1     FSMFKQRKLVKRHAAEIFTSIAIASTFSLYSTAILGRVIGLEPSLTISILPRCITVALAL     419
OsPLGG2     FSMFNQRKLVRRHAAEIFTSIAVASTFSLYSTAILGRVVELEPILTISILPRCITVALAL     346
            ****:*****:*********: :::.*******::**:: *** **:*************

AtPLGG1     SIVSLFEGTNSSLTAAVVVVTGLIGANFVQVVLDKLRLRDPIARGIATASSAHGLGTAAL     472
OsPLGG1     SIVSFFEGVNSSLTAAVVVLTGLIGANFVQAAMDKLGLNDPIARGIGTASSAHGLGTAAL     479
OsPLGG2     RVVSLFEGVNTSVTAAVVVLTGLIGANFAQAVMDKLRLKDPIARGIGTASSAHGLGTAAV     406
             :**:***.*:*:******:********.*..:*** *.*******.************:

AtPLGG1     SAKEPEALPFCAIAYALTGIFGSLLCSVPAVRQSLLAVVG------------     512
OsPLGG1     SAKEPEALPFCAIAYGLTGIFGSLICSVPAVRQSLVFIAG------------     519
OsPLGG2     SAKEPEALPFCAIAYALTGVVASLFCSLPAIRHSLVFIAGDASASQTQHFSY     458
            ***************.***:..**:**:**:*:**: :.*
```

Supplemental Figure S1. Protein sequence alignment of AtPLGG1 and rice candidates. Protein sequences of two rice homologs, OsPLGG1 (LOC_Os01g32830) and OsPLGG2 (LOC_Os10g42780), were aligned with that of AtPLGG1 (At01g32080). Green color indicates a predicted chloroplast targeting peptide.

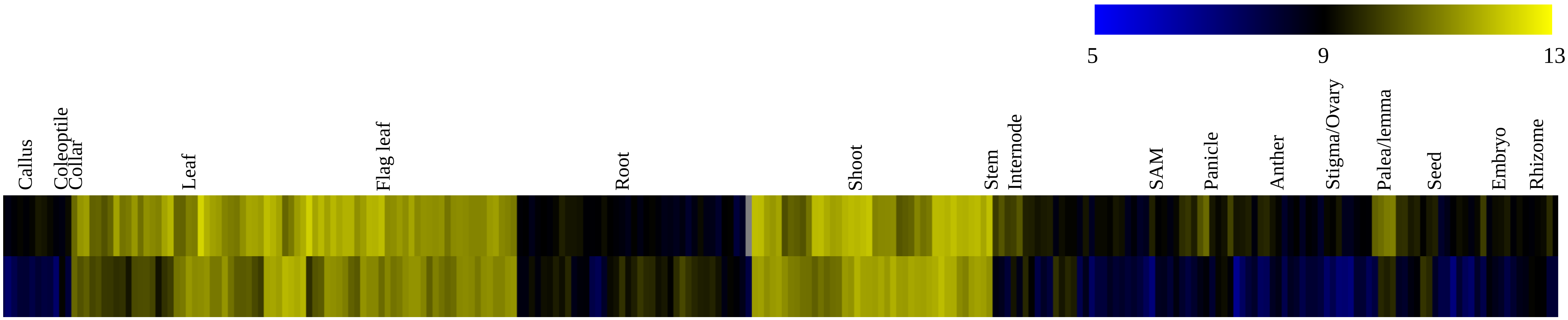

Supplemental Figure S2. Heat map expression analysis of OsPLGG1 (top) and OsPLGG2 (bottom) using Affymetrix rice microarray data downloaded from NCBI GEO.

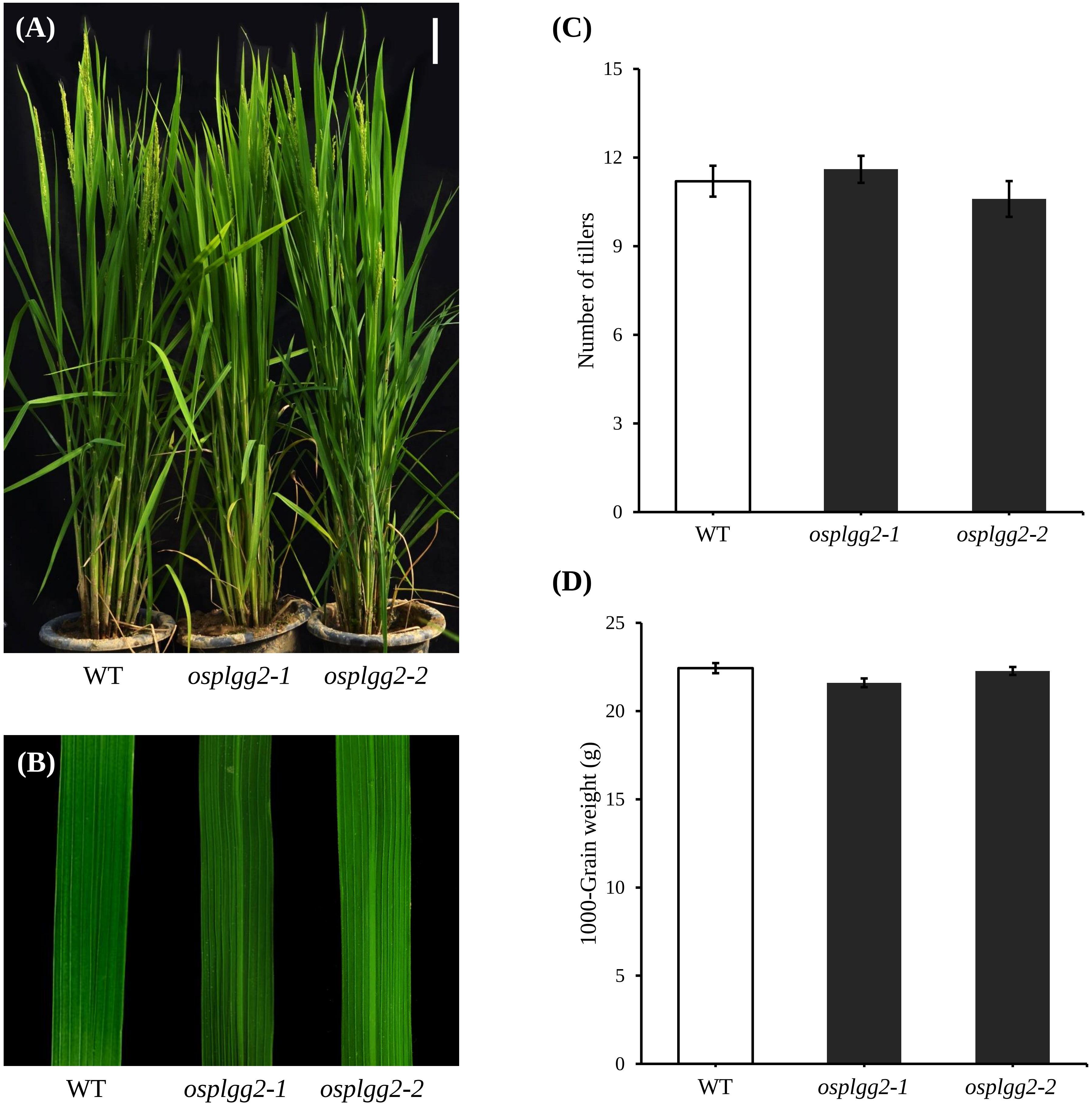

Supplemental Figure S3. Phenotypic analysis of *osplgg2* mutants. **(A, B)** Mature plants **(A)** and leaf blades **(B)** of WT, *osplgg2-1* and *osplgg2-2* mutant plants grown for three months in the paddy field. Bar = 10 cm. **(C, D)** Number of tillers **(C)** and 1000-grain weight **(D)** of WT, *osplgg2-1* and *osplgg2-2* plants. The experiment consisted of five independent plants per each line. Error bars indicate SEM. $P > 0.05$ according to Student's *t*-tests.

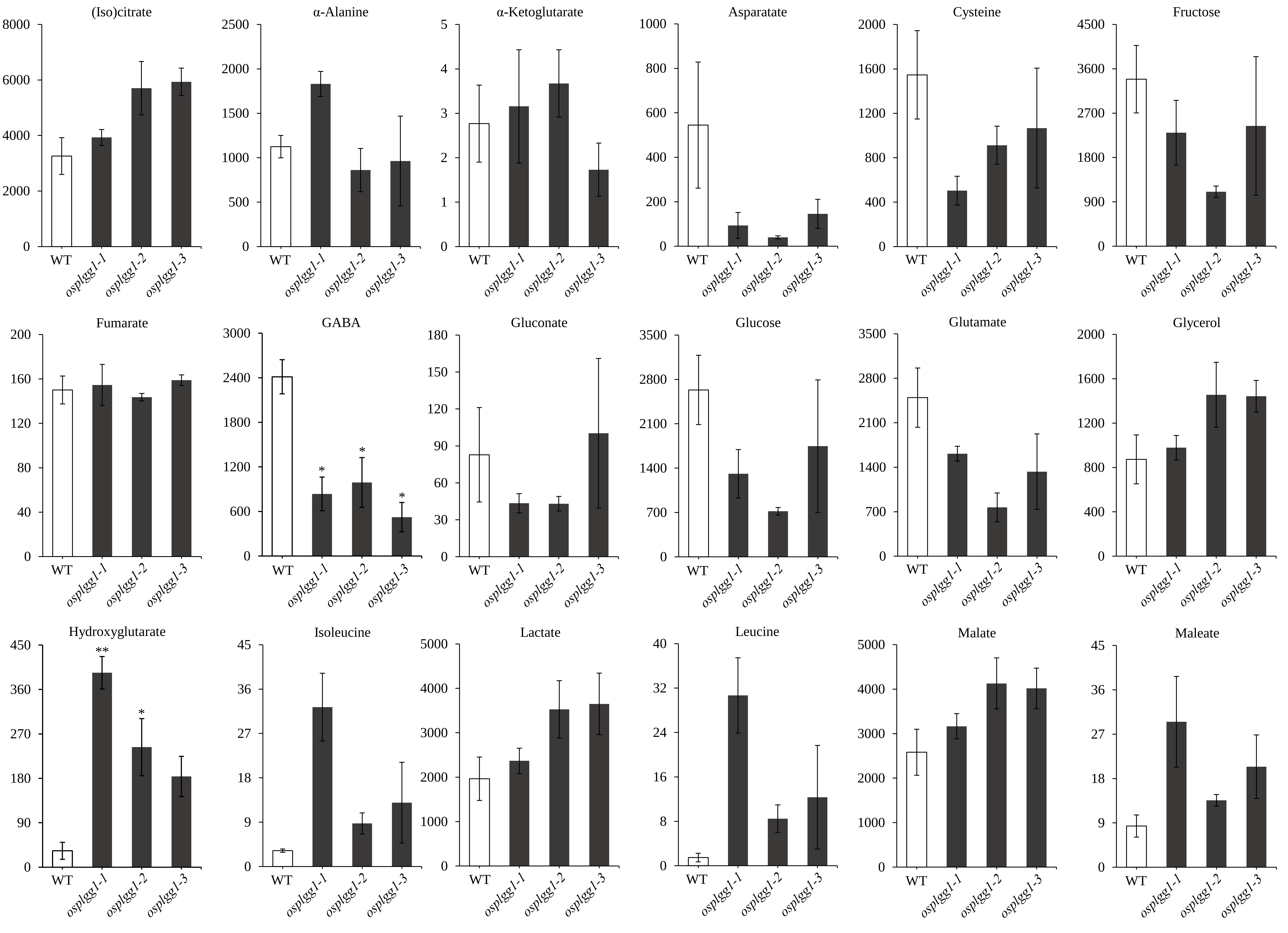

(Iso)citrate
α-Alanine
α-Ketoglutarate
Asparatate
Cysteine
Fructose
Fumarate
GABA
Gluconate
Glucose
Glutamate
Glycerol
Hydroxyglutarate
Isoleucine
Lactate
Leucine
Malate
Maleate
WT
osplgg1-1
osplgg1-2
osplgg1-3
*
**

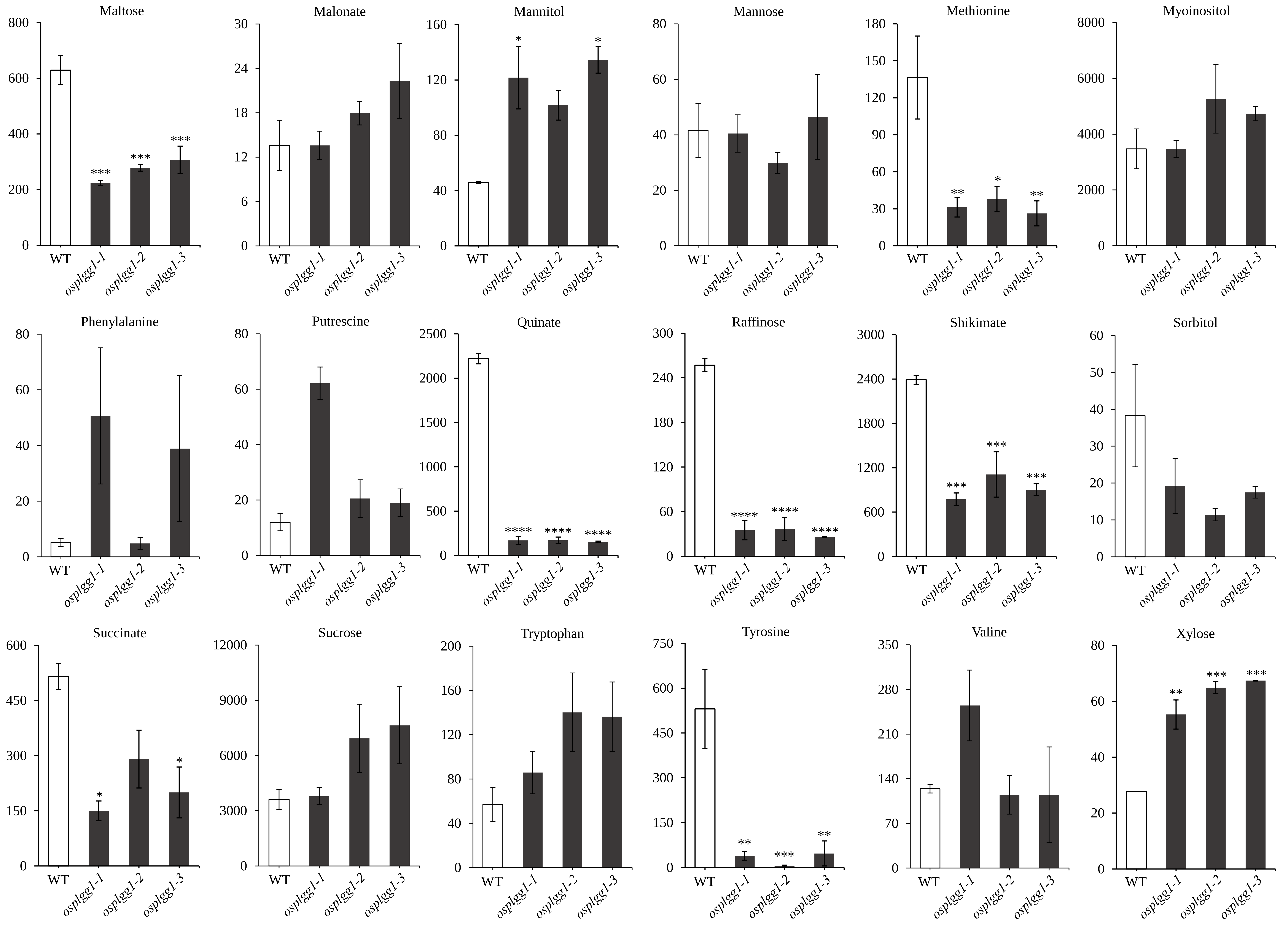

Maltose
0
200
400
600
800
***
***
***
WT
osplgg1-1
osplgg1-2
osplgg1-3
Malonate
0
6
12
18
24
30
Mannitol
0
40
80
120
160
*
*
Mannose
0
20
40
60
80
Methionine
0
30
60
90
120
150
180
**
*
**
Myoinositol
0
2000
4000
6000
8000
Phenylalanine
0
20
40
60
80
Putrescine
0
20
40
60
80
Quinate
0
500
1000
1500
2000
2500
****
****
****
Raffinose
0
60
120
180
240
300
****
****
****
Shikimate
0
600
1200
1800
2400
3000
***
***
***
Sorbitol
0
10
20
30
40
50
60
Succinate
0
150
300
450
600
*
*
Sucrose
0
3000
6000
9000
12000
Tryptophan
0
40
80
120
160
200
Tyrosine
0
150
300
450
600
750
**
***
**
Valine
0
70
140
210
280
350
Xylose
0
20
40
60
80
**
***
***

Supplemental Figure S4. Relative abundances of metabolites which are not intermediates of photorespiration in leaves of one-month-old WT and *osplgg1* mutant plants grown in the paddy field. The relative metabolite levels were normalized to an internal standard (ribitol) and the fresh weight of the samples. Three independent biological replicates were used in the experiment, each having three technical replicates. Error bars indicate SEM. Student's *t*-test was used to show statistical differences. *P < 0.05; **P < 0.01; ***P < 0.001; ****<0.0001.
