# Supplementary material for: Loss of Function of Rice Plastidic Glycolate/Glycerate Translocator 1 Impairs Photorespiration and Plant Growth
Source: Front Plant Sci. 2020 Jan 24;10:1726. doi: 10.3389/fpls.2019.01726 (PMC6993116; doi:10.3389/fpls.2019.01726)
Supplement: Supplementary file 3 [file Table_1.docx]

**Table S1.** Primers used in this study.

| Primers | Sequences |
| --- | --- |
| G1F | 5’-ACCGGTTCTGACTTGATATTACCTTTTGT-3’ |
| G1R | 5’-CGAGCTATAAAATCCATACATAGCTCACG-3’ |
| G2F | 5’-GTATGATTTGAAATAATAGATGGCGCATG-3’ |
| G2R | 5’-TTAGAGTTGATGTTGAAGTTTTCTCAAGT-3’ |
| T1 | 5’-GTGGTGCAGATGAACTTCAGGGTC-3’ |
| T2 | 5’-CTAGAGTCGAGAATTCAGTA-3’ |
| T3 | 5’-ATCCAGACTGAATGCCCACAGG-3’ |
| RT1F | 5’-CAACATTCTCGTTATACTCAACTG-3’ |
| RT1R | 5’-ACAAACAAAAACAAAACTGATGTA-3’ |
| RT2F | 5’-CTGTGGCATCAACATTCTCGTTAT-3’ |
| RT2R | 5’-GAAGCTTGTCCATCACTGCTTG-3’ |
| UBQ5 F | 5’-GACTACAACATCCAGAAGGAGTC-3’ |
| UBQ5 R | 5’-TCATCTAATAACCAGTTCGATTTC-3’ |
| MF | 5’-ATGTATGGATTTTATAGCTCGTCGG-3’ |
| MR | 5’-TGCACAACAAATGATTGGGTGAA-3’ |
| FLCF | 5'-AGAAGGAGAAGAAGAGAAGAGAAG-3' |
| FLCR | 5'-TATTACAACGGAGACATAATCACA-3’ |
